# Manualised Cognitive Behaviour Therapy for Anorexia Nervosa: Use of Treatment Modules in the ANTOP Study

**DOI:** 10.3390/jcm7110398

**Published:** 2018-10-29

**Authors:** Gaby Resmark, Brigid Kennedy, Maria Mayer, Katrin Giel, Florian Junne, Martin Teufel, Martina de Zwaan, Stephan Zipfel

**Affiliations:** 1 Department of Psychosomatic Medicine and Psychotherapy, University Hospital Tübingen, 72076 Tübingen, Germany; brigid.kennedy@med.uni-tuebingen.de (B.K.); maria.mayer@med.uni-tuebingen.de (M.M.); katrin.giel@med.uni-tuebingen.de (K.G.); florian.junne@med.uni-tuebingen.de (F.J.); stephan.zipfel@med.uni-tuebingen.de (S.Z.); 2 School of Psychology, The University of Sydney, Camperdown 2006, Australia; 3 Department of Psychosomatic Medicine and Psychotherapy, University of Duisburg-Essen, 45147 Essen, Germany; martin.teufel@lvr.de; 4 Department of Psychosomatic Medicine and Psychotherapy, Hannover Medical School, 30625 Hannover, Germany; dezwaan.martina@mh-hannover.de

**Keywords:** anorexia nervosa, cognitive behaviour therapy, manualised treatment, modules, worksheets

## Abstract

Standardised treatment manuals facilitate therapy planning and enhance comparability for research purposes. Within the Anorexia Nervosa Treatment of Out Patients (ANTOP) study, the largest multisite outpatient intervention trial in anorexia nervosa (AN) to date, manualised enhanced cognitive-behavioural therapy (CBT-E) was offered as one treatment modality. The manual consisted of 9 modules, of which *Motivation, Nutrition, Formulation and Relapse Prevention* were compulsory. Homework worksheets were provided, to ensure the transfer of therapeutic improvements to daily life. This study investigated the use of modules and worksheets in order to explore practice styles of trained therapists in the treatment of AN. This secondary analysis was based on log-sheets (*n* = 2604) CBT-E therapists completed after each session. Frequencies of modules and worksheets used across all sessions were calculated. Relationships, such as that between use of module and duration of illness, were examined. The most commonly used module was *Motivation*. In patients with longer illness duration, the module *Self Esteem* seemed to be particularly important. The worksheet *Scales*, balancing the pros and cons of AN, was prioritised by therapists. The results underline the importance of motivational work in the treatment of AN, including validating the ambivalence experienced by most AN patients. With increasing duration of illness, resource-oriented elements, such as self esteem stabilisation, should be of focus.

## 1. Introduction

Treatment manuals for psychotherapeutic interventions guarantee that the path of treatment remains relatively focused, ensuring a standardised quality of therapy [1]. The clear structure associated with manualised treatment also increases transparency associated with the treatment, and with that, patient motivation [2]. Yet, the use of treatment manuals continues to be a controversial discussion point in the psychotherapy field [2]. Critics argue that use of manuals leads to rigid treatment, which neglects individual components of the patient’s disorder, and reduces scope for therapist innovation [1,2].

Manuals exist for a vast array of treatments, including cognitive behaviour therapy (CBT) [3,4,5]. A convincing bank of evidence suggests that manualised CBT is effective in treating eating disorders [6,7,8]. Studies have shown that CBT can produce weight gain in anorexia nervosa (AN) patients [9], as well as improvements in eating disorder pathology for patients with AN [9], bulimia nervosa and eating disorders not otherwise specified [10]. A recently developed modified version of CBT, called enhanced cognitive behaviour therapy (CBT-E) [3], employs a transdiagnostic approach addressing all eating disorders. CBT-E has been shown to produce lasting improvements in body-mass index and eating disorder pathology in AN patients [11], and to be equally as effective as other “standard” treatment options [11,12].

One aspect which warrants consideration is the inclusion of modules in CBT treatment manuals. Modules are a form of building block for the treatment [4]; they outline the focal points of the planned treatment and seek to guide the therapy. Several modular CBT manuals have been developed to treat eating disorders; often, these manuals provide a wide selection of modules for therapists and patients to choose from [3,4,5,13]. Worksheets are another element often included as a supplementary resource to accompany modules; for example, in Wilhelm et al.’s CBT manual for body dysmorphic disorder [5] and in Legenbauer and Vocks’ manual for anorexia and bulimia treatment [4]. Legenbauer and Vocks’ manual [4], only available in German, is a practical manual designed for the cognitive behavioural treatment of eating disorders. The manual contains chapters addressing specific concepts related to eating disorders, such as motivation. Each chapter is accompanied by specific activities and detailed worksheets to assist the therapy. Worksheets can be used in-session and administered as homework, and facilitate the transfer of therapeutic progress into daily life. Similarly, Fairburn’s CBT-E manual considers “Next Steps”, an alternative term for homework, to be an integral component of treatment [3]. Most CBT manuals provide a rather clear structure, but should be seen as a guide, rather than an inflexible, predetermined protocol [14].

While past research has demonstrated that modular treatment manuals can produce positive treatment effects in eating disorder patients, to the authors’ knowledge, no research has investigated the actual *implementation* of such manuals. Hence, there is an absence of research investigating the ways in which therapists execute manualised treatment. This study aimed to tackle this research gap, by investigating the practice styles of therapists administering a manualised CBT-E treatment to outpatients with AN. The manual of focus, written in German, was developed in 2007, prior to the publication of Fairburn’s CBT-E manual [3]. It was written based on a 2-day workshop delivered by Fairburn. The manual was designed specifically for the ANTOP (Anorexia Nervosa Treatment of Out Patients) study, a German multisite randomised control trial in outpatients with AN [15,16]. The design and main outcome of the ANTOP study have been published elsewhere [15,16]. The present study sought to answer the following research questions:

- What were the most commonly used modules?
- What were the most commonly used worksheets?
- Was there a relationship between stage of therapy and module used?
- Was there a relationship between duration of illness and module used?

## 2. Methods

### 2.1. CBT-E in the ANTOP Study

This study was conducted as a secondary analysis of data from the ANTOP study. In one arm of this study, AN patients received 40 individual sessions of CBT-E over 10 months. Therapy was categorised into three stages: stage 1 (sessions 1–16) involved therapy twice a week for 2 months, stage 2 (sessions 17–32) involved therapy once a week for 4 months, and stage 3 (sessions 33-40) involved therapy once every 2 weeks for 4 months. Twenty-four CBT therapists, trained initially by Fairburn, used the specifically designed CBT-E ANTOP manual to guide treatment. The manual contained 9 modules, 4 of which were compulsory (Table 1). Worksheets were also provided for optional use during sessions and as homework. At the time of the ANTOP manual development, Fairburn’s available material did not contain any worksheets, and hence, worksheets were taken from Legenbauer and Vocks’ German CBT manual [4]. At the end of each therapy session, therapists were required to fill out a log-sheet, documenting detailed information regarding the content of the session. Although several modules could be used per session, therapists were required to record the single module which was the main focus throughout that session. Therapists could also record up to 2 worksheets given to the patient in the session, or as homework. This information provided the data for the current analysis.

### 2.2. Sample

Of the eighty AN patients assigned to the CBT-E arm in the ANTOP study, 78 commenced treatment and 65 completed treatment (that is, they attended at least 27 of the 40 sessions). Only female, adult patients (aged ≥ 18 years) were included in the ANTOP study. When the study commenced, patients’ mean age was 27.4 years and mean body-mass index (kg/m^2^) was 16.82. Forty-nine patients (61%) had an illness duration of less than or equal to 6 years, and 31 (39%) had AN for longer than 6 years [16]. The data used for this secondary analysis comprised of 2604 session logs; this number was less than the total possible number of session logs (3120) as not all patients completed all 40 treatment sessions. Only logs which contained relevant information regarding the component of interest were included in each analysis.

The ANTOP study was approved by the ethics board of the faculty of medicine, University Hospital Tübingen, on the 21/02/2006 (ref: 440/2006). Additionally, the study was approved by the ethics committees at each of the participating treatment centres. All procedures performed in studies involving human participants were in accordance with the ethical standards of the institutional and/or national research committee and with the 1975 Helsinki declaration and its later amendments or comparable ethical standards.

### 2.3. Statistics

All statistical analyses were conducted using IBM SPSS Statistics version 25 (IBM, Armonk, NY, USA). Frequency tests were conducted in order to identify the most commonly used modules and worksheets across all CBT-E sessions. Crosstabs were displayed in order to investigate the relationship between choice of module and stage of therapy. An overall chi square test of independence was conducted to determine the relationship between module and duration of illness, followed by post hoc standardised residuals testing (absolute value greater than 2.00 indicated significance [17]). Patients were classified into two groups: those with an illness duration of equal to or less than 6 years, and those with an illness longer than 6 years. Duration of illness was classified in this way, because in the ANTOP study the randomisation had been stratified according to this dichotomised variable.

## 3. Results

### 3.1. Modules

**Figure 1:**
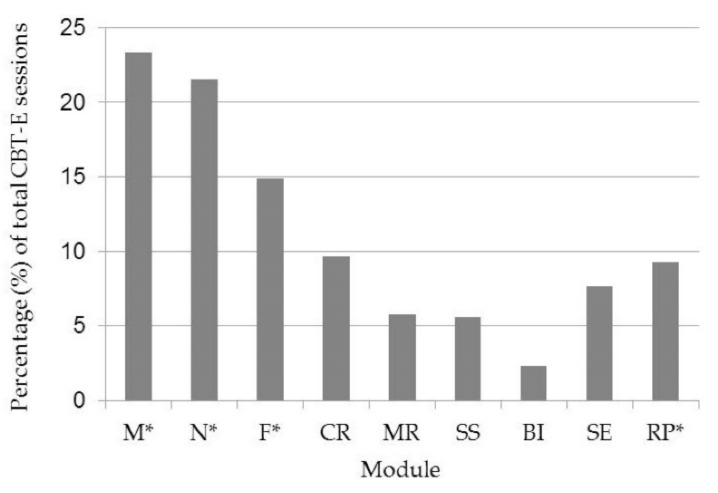
Frequency (percentage) of focus modules (*n* = 2411) used throughout treatment. M = Motivation, N = Nutrition, F = Formulation, CR = Cognitive Restructuring, MR = Mood Regulation, SS = Social Skills, BI = Body Image, SE = Self Esteem, RP = Relapse Prevention. Asterisks represent compulsory modules.

Across all 2604 CBT-E sessions, the focus module was recorded a total of 2411 times. Figure 1 depicts frequencies of module use.

### 3.2. Relationship between Module and Stage of Therapy

**Figure 2:**
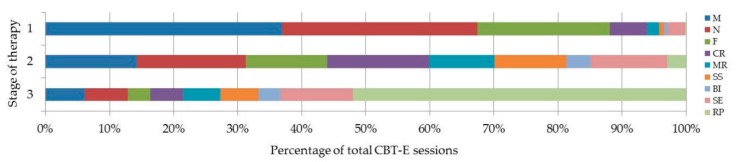
Relationship between frequency (percentage) of applied modules (*n* = 2411) and stage of therapy. M = Motivation, N = Nutrition, F = Formulation, CR = Cognitive Restructuring, MR = Mood Regulation, SS = Social Skills, BI = Body Image, SE =Self Esteem, RP =Relapse Prevention. Stage 1: sessions 1–16, Stage 2: sessions 17–32, Stage 3: sessions 33–40.

As can be seen in Figure 2, stage of therapy appeared to influence choice of module. While Stage 1 sessions focused on modules such as *Motivation* and *Nutrition* most frequently, over 50% of Stage 3 sessions focused on *Relapse Prevention*.

### 3.3. Relationship between Module and Duration of Illness

There was a statistically significant relationship between choice of module and duration of illness, *X*² (8, *n* = 2411) = 15.937, *p* = 0.043. Post-hoc tests analysing standardised residuals revealed that differences between the duration of illness groups lay in the use of the *Self Esteem and Resources* module; this module was used significantly more often with patients who had a duration of illness longer than 6 years, compared to those who had an illness duration of 6 years or less.

### 3.4. Worksheets

The use of worksheets was recorded 888 times across all session logs. 590 additional records of worksheet use were excluded from analysis, as they did not refer to any of the 55 worksheets made available to the therapists. A list of the top ten most commonly used worksheets was generated using frequency analysis (Table 2). 

*The Scales* was the most commonly used worksheet, distributed 107 times (12%). This worksheet involved patients recording the short- and long-term pros and cons of their eating disorder on either side of a balance scale. Next to each pro or con, they were instructed to write a number from 1 to 100, indicating how important they considered this factor to be. After completing their list, patients were asked to consider both the pros and cons lists in their entireties and to assign each side of the scale a number. This number was to be used as a measure of whether the eating disorder is more friend or foe.

*Family relationships* was the second most commonly used worksheet, distributed 56 times (6.3%). This worksheet involved patients reflecting on the relationships within their family, specifically throughout their pubescent years, or whichever period they feel was most important for the development of their eating disorder. Patients were instructed to draw or write the names of their family members (including themselves) within a rectangle. They were told to use lines to connect the family members; normal lines represented positive and stable relationships, and dotted lines represented relationships characterised by conflict. On the left of the page, in the smaller boxes, patients listed the distinctive traits they believe characterise each of their family members. The general aim of this worksheet was to encourage reflection on family relationships, and also to identify factors potentially influencing the emergence and persistence of the disorder. For information on further worksheets, see Legenbauer and Vocks’ Manual.

## 4. Discussion

This secondary analysis of data from the ANTOP study provides insight into the practice styles of experienced therapists administering manualised CBT-E to outpatients with AN. It sheds light on which modules and corresponding worksheets were most commonly used. Additionally, it considers the use of modules in more depth, in particular in relation to stage of therapy and duration of illness.

The four most commonly used modules overall were *Motivation, Nutrition, Formulation* and *Cognitive Restructuring,* suggesting that they are arguably the most important modules of focus during CBT-E for AN patients. It is, however, important to acknowledge that three of these four modules were indeed compulsory modules, meaning they were required to be used for at least 5 of the 40 sessions. The fact that focus was given more often to the optional module *Cognitive Restructuring* than to the compulsory module *Relapse Prevention* can be interpreted in two ways. On the one hand, cognitive restructuring represents an essential strategy within CBT. On the other hand, just over a quarter of the ANTOP sample still had full syndrome AN at the end of treatment [16]; this might have resulted in less use of *Relapse Prevention*, as use of this module often assumes absence of symptoms. It should also be acknowledged that although module choice was oriented around patient needs, choice of module would have also been influenced by the therapists’ practice styles and beliefs.

The *Motivation* and *Nutrition* modules were of the greatest focus, both being distributed in over 20% of sessions. These findings coincide with the extensive bank of literature suggesting that these aspects should be of pivotal focus throughout AN treatment. The ego syntonic nature of the illness often contributes to an ambivalence to change in AN patients [18]; the symptoms which characterise the illness, such as dangerously low body weight, are unfortunately characteristics which patients value, and hence often wish to maintain. Indeed, patients with AN rarely seek treatment on an entirely voluntary basis [18]. There is evidence that a patient’s motivation to change is arguably one of the strongest predictors of treatment success [19,20]. Nutrition is another pivotal aspect to successful CBT-E treatment of AN as patients need to normalise their eating behaviour and restore weight in order to overcome the illness [21,22]. Additionally, evidence suggests that with better nutrition, cognitive functioning can be improved, and subsequently, responsiveness to interventions [23].

The limited use of the *Body Image* module also warrants consideration. Disturbed body image is one of the characterising features of AN [24,25,26,27]. Indeed, body image distortion is not only a predictor of the development of AN [25], but also for the longevity of the diagnosis, and likelihood of relapse [24]. Perceptions of body image have also been found to be significantly related to depression and anxiety symptoms in AN patients [26]. It is, therefore, clear that body image is intimately intertwined with AN, and should accordingly be of pivotal focus in treatment. Yet, the current data analysis revealed that the *Body Image* module was surprisingly the least frequently used module among the ANTOP therapists; specifically, it was used in less than 3% of sessions. The current findings provide evidential support for the concern that body image disturbances are somewhat neglected in eating disorder therapy [4,28]. Accordingly, treatment outcomes might be improved by placing more focus on body image disturbances [25]. Research comparing two versions of CBT for eating disorder patients revealed that treatment which specifically addressed body image disturbance produced greater improvements at end of treatment and one year follow up, than CBT treatment which did not [29]. When considered in conjunction with this research, the current findings highlight a potential limitation in current treatment practice; that therapists are perhaps not placing enough emphasis on the concept of body image in their treatment of AN patients.

Data analysis found that during stage 1 (sessions 1–16) therapy sessions, the *Motivation* and *Nutrition* modules were of predominant focus. These findings are in line with existing literature [3,30], which suggests that in order for therapy to be successful, initial stages of therapy should address aspects such as patient’s motivation to engage in therapy, healthy eating habits, and weight gain. Analysis also revealed that over 50% of stage 3 sessions focused on the *Relapse Prevention* module. This again coincides with the literature, which suggests that in order to ensure long-term results, final stages of treatment should address how progress can be maintained upon cessation of therapy, and how relapse can be prevented [3,30]. Overall, these results suggest that therapists administering CBT-E in the ANTOP study were administering therapy in a manner that is in accordance with the suggested progression of AN treatment.

There was a significant relationship between duration of illness and choice of modules, specifically in the use of the *Self Esteem* *and Resources* module. This module was significantly more likely to be used for patients who had an illness duration of longer than 6 years. Current research suggests that although some patients do recover fully from AN, approximately 20% of patients go on to develop a severe and enduring form of the disorder that is resistant to treatment [31]. Touyz and colleagues argue that this subset of patients have a unique set of needs, different to those of other AN patients, and hence suggest that a different approach should be taken in their treatment [32,33]. This approach, closely linked to the Recovery Model [32], does not view recovery simply as an absence of symptoms. Instead, focus is shifted away from weight gain towards aspects such as improving quality of life and hope for the future [33]. The findings from the current study seem to compliment this alternative approach. In their treatment of longer suffering AN, CBT-E therapists of the ANTOP study seemed to deem it necessary to place greater focus on resource-oriented aspects outside of the AN symptomatology, such as the *Self Esteem and Resources* module. 

The data analysis also revealed which of the available 55 worksheets were used most frequently. In accordance with the priority given to the *Motivation* module, the worksheet *The Scales,* addressing the pros and cons of the eating disorder, was used almost double the amount of times than any other worksheet. *Family Relationships,* which required patients to construct a family diagram, was the second most frequently used worksheet. The frequent use of these worksheets highlights the importance of these specific topics of motivation and relationships throughout the treatment process. 

### Limitations and Future Research

While the design and conduct of the ANTOP study followed the highest aspirations of international standards in clinical research, this subproject was limited due to its nature as a secondary analysis. Consequently, the results must be considered as hypotheses to be tested in future studies. It was a completer analysis, meaning only data provided by patients currently in treatment could be considered. Additionally, the categorical nature of the data limited the scientific quality of the analysis; mainly descriptive analysis was provided. Finally, although this study included an in-depth analysis of the practice styles of experienced therapists, it did not assess the effectiveness of these practice styles. Future research would therefore benefit from investigating whether use of certain modules, or combinations of modules, results in better treatment outcomes for AN patients. Furthermore, it could be useful to investigate whether certain worksheets were more commonly used in younger patients, as many of them were designed in a “girlish” way (e.g., the worksheet *My Strengths* in the *Self Esteem* module was pink and decorated with flowers), and so therapists may have chosen not to give them to older patients.

## 5. Conclusions

To the authors’ knowledge this is the first scientific analysis which explores the practical application of a modular therapy manual in the treatment of eating disorders. Analysing individual outpatient therapy administered by experienced therapists involved in the ANTOP study, this paper provides other therapists with practical recommendations regarding the use of modules and corresponding worksheets within manualised CBT-E for AN. Analysis revealed that *Motivation*, *Nutrition*, *Formulation* and *Cognitive Restructuring* were the most common modules of focus. In particular, *Motivation* and *Nutrition* seemed to be most relevant during the initial stages of treatment, whereas *Relapse Prevention* was more relevant in the final treatment stage. The module *Self Esteem* appeared to be particularly relevant for patients who had a duration of illness longer than 6 years, a finding which complements recent research advocating a so-called recovery model for long-term sufferers of AN. *Body Image* was often neglected; a concerning finding, in light of recent research highlighting the importance of addressing body image disturbances in AN patients. The most commonly used worksheet was *The Scales* within the module *Motivation.* These findings underline the importance of actively addressing the ambivalence often present in patients with AN in order to facilitate readiness for change. Furthermore, the findings show clearly that modern manualised CBT is much more than teaching strategies and techniques. It should leave room to address themes that might maintain the individual’s eating disorder and therefore need to be solved in order to allow for recovery.

## Figures and Tables

**Table 1:** Modules in the Anorexia Nervosa Treatment of Out Patients (ANTOP) study CBT-E manual.

Module	Module Content
**Compulsory Modules**	
Motivation (Starting Well)	Building a therapeutic relationship, reflecting on pros and cons of anorexia nervosa (AN), and discussing healthy eating behaviours
Nutrition	Establishing and maintaining a regular healthy eating pattern
Formulation	Understanding what causes and maintains the individual’s eating disorder
Relapse Prevention (Ending Well)	Maintaining positive behavioural changes learnt throughout the course of therapy and preparing to cope with setbacks
**Optional Modules**	
Cognitive Restructuring	Learning to challenge dysfunctional beliefs concerning eating, weight and the body
Mood Regulation	Recognising and coping with negative emotions
Social Skills	Improving communication and conflict resolutions skills
Body Image	Addressing the negative attitudes towards patients’ own bodies, and the influence of perceived figure/weight on self-worth
Self Esteem and Resources	Increasing self-worth: Identifying strengths, establishing new hobbies and interests, reflecting on what brings happiness

**Table 2:** Top ten distributed worksheets, listed in descending order according to frequency used (*n* = 888).

Name of Worksheet (Module)	Number of Times Distributed (%)
The Scales (Motivation)	107 (12)
Family relationships (Formulation)	56 (6.3)
Two letters to the eating disorder (Motivation)	53 (6.0)
How I’d like to change my eating behaviour (Nutrition)	43 (4.8)
Cognitive distortions (Cognitive Restructuring)	37 (4.2)
What have I learnt (Formulation)	34 (3.8)
Analysis of a monitoring record (Nutrition)	29 (3.3)
Paths to change (Nutrition)	27 (3.0)
Toolbox for emergencies (Relapse Prevention)	26 (2.9)
What I need to be content (Self Esteem)	26 (2.9)
